# Bilateral Pachychoroid disease with type 3 Uveal effusion syndrome in one eye and central serous Chorioretinopathy in contralateral eye: a case report

**DOI:** 10.1186/s12886-022-02316-y

**Published:** 2022-02-23

**Authors:** Hajime Onoe, Hiroyuki Shimada, Akiyuki Kawamura, Hiromi Hirosawa, Koji Tanaka, Ryusaburo Mori, Hiroyuki Nakashizuka

**Affiliations:** Department of Ophthalmology, School of Medicine, Nihon University Hospital, 1-6 Surugadai, Kanda, Chiyodaku, Tokyo, 101-8309 Japan

**Keywords:** Case report, Central serous chorioretinopathy, Full-thickness sclerectomies, Pachychoroid, Type 3 uveal effusion syndrome

## Abstract

**Background:**

We report a case of bilateral pachychoroid disease with type 3 uveal effusion syndrome (UES) in one eye and central serous chorioretinopathy (CSC) in the contralateral eye.

**Case presentation:**

A 65-year-old man presented to our department because of decreased vision. Visual acuity was 16/20 in the right eye and 2/20 in the left eye, with normal axial lengths. The left eye was diagnosed with CSC and underwent photocoagulation 8 years ago. The right eye showed inferior non-rhegmatogenous retinal detachment and peripheral choroidal detachment. Macular optical coherence tomography showed submacular fluid in the right eye, pachychoroid in both eyes, and choroidal thickness of 565 μm in the right and 545 μm in the left eye. In both eyes, fluorescence angiography showed window defects and mild fluorescence leakage, and indocyanine green angiography showed dilated choroidal vessels, mild choroidal hyperpermeability, and mild dye leakage. The left eye was diagnosed with chronic CSC. Initially, chronic CSC was also suspected in the right eye. However, photodynamic therapy failed, with worsened retinal detachment and visual acuity. Pachychoroid in the peripheral fundus (choroidal thickness 820 μm) was observed only in the right eye. Based on these findings, UES was diagnosed in the right eye. Sclerectomies were performed. The absence of scleral thickening and glycosaminoglycan deposition led to a final diagnosis of type 3 UES. The procedure was not effective, due to connective tissue regeneration at the sclerectomy sites. In the revision surgery, mitomycin-C was used with sclerectomies. One month after surgery, retinal and choroidal detachment disappeared, visual acuity recovered to 8/20, pachychoroid in the macula and peripheral fundus decreased, and choroidal thickness decreased to 352 μm in the macula and 554 μm in inferior peripheral fundus.

**Conclusions:**

Pachychoroid in the posterior pole was the common finding in type 3 UES and CSC, although extensive pachychoroid in the peripheral fundus may have caused retinal and choroidal detachment in the eye with type 3 UES. Full-thickness sclerectomies with mitomycin-C improved pachychoroid in the peripheral fundus and resolved retinal and choroidal detachment, clearly indicating that the sclera was the main cause of type 3 UES.

## Background

In 1963, Schepens and Brockhurst [1] described non-rhegmatogenous retinal detachment as a specific type of uveal effusion. In 1975, Brockhurst [2] described nanophthalmic uveal effusion, in which the thick sclera associated with small eyes compresses the vortex veins, resulting in retinal and choroidal detachment. In 1982, Gass and Jallow [3] reported uveal effusion syndrome (UES) as a disease entity of idiopathic detachment of the choroid, ciliary body, and retina.

Since 1982, UES has been classified into two types: nanophthalmic UES and idiopathic UES. The classification of Uyama et al. in 2000 [4] divided idiopathic types into type 2 UES and type 3 UES, and this classification is currently used worldwide. In type 2 UES, the eyeball size is normal with scleral abnormalities, and in type 3 UES, the eyeball size is normal with no scleral abnormalities.

In the report of Uyama et al. [4], two eyes (two patients) that showed no scleral thickening and no glycosaminoglycan deposition were classified as type 3 UFS, and subscleral sclerectomies (sclerectomies under the scleral flap) were not effective in these eyes. Since no reoperation was performed, the cause of the ineffective subscleral sclerectomies was unknown. Terubayashi et al. [5] reported a case of type 3 UES in which sclerotomies were not effective, and vitrectomy, phacoemulsification, and silicone oil tamponade were performed with favorable outcomes. Thus, in previously reported cases of type 3 UES, when subscleral sclerectomies or sclerotomies were not effective, the cause of recurrence was unknown because scleral surgeries were not performed again.

We report a case of bilateral pachychoroid disease with type 3 UES in one eye and chronic central serous chorioretinopathy (CSC) in the contralateral eye. Both eyes showed similar findings in the posterior pole on fluorescein angiography (FA), indocyanine green angiography (ICGA), and optical coherence tomography (OCT). In the eye with CSC, the lesions were mainly localized in the macula and photocoagulation was conducted. In the eye with type 3 UES in which retinal and choroidal detachment occurred, full-thickness sclerectomies alone were not effective, but mitomycin-C with full-thickness sclerectomies achieved favorable outcomes.

## Case presentation

A 65-year-old man presented to our hospital with chief complaint of decreased vision in his right eye. Eight years ago, his left eye was diagnosed with CSC and treated with conventional focal photocoagulation in another hospital, but relapsed a few months later. A systemic disease workup excluded rheumatic diseases and other systemic diseases.

His corrected visual acuity was 16/20 in the right eye and 2/20 in the left eye. Axial length was 22.44 mm in the right and 22.52 mm in the left. Intraocular pressure (IOP) was 11 mmHg in the right and 17 mmHg in the left. Shallow anterior chamber was seen in both eyes; 2.17 mm in the right and 2.55 mm in the left, but there were no inflammatory cells in the anterior chamber and vitreous.

**Fig. 1:**
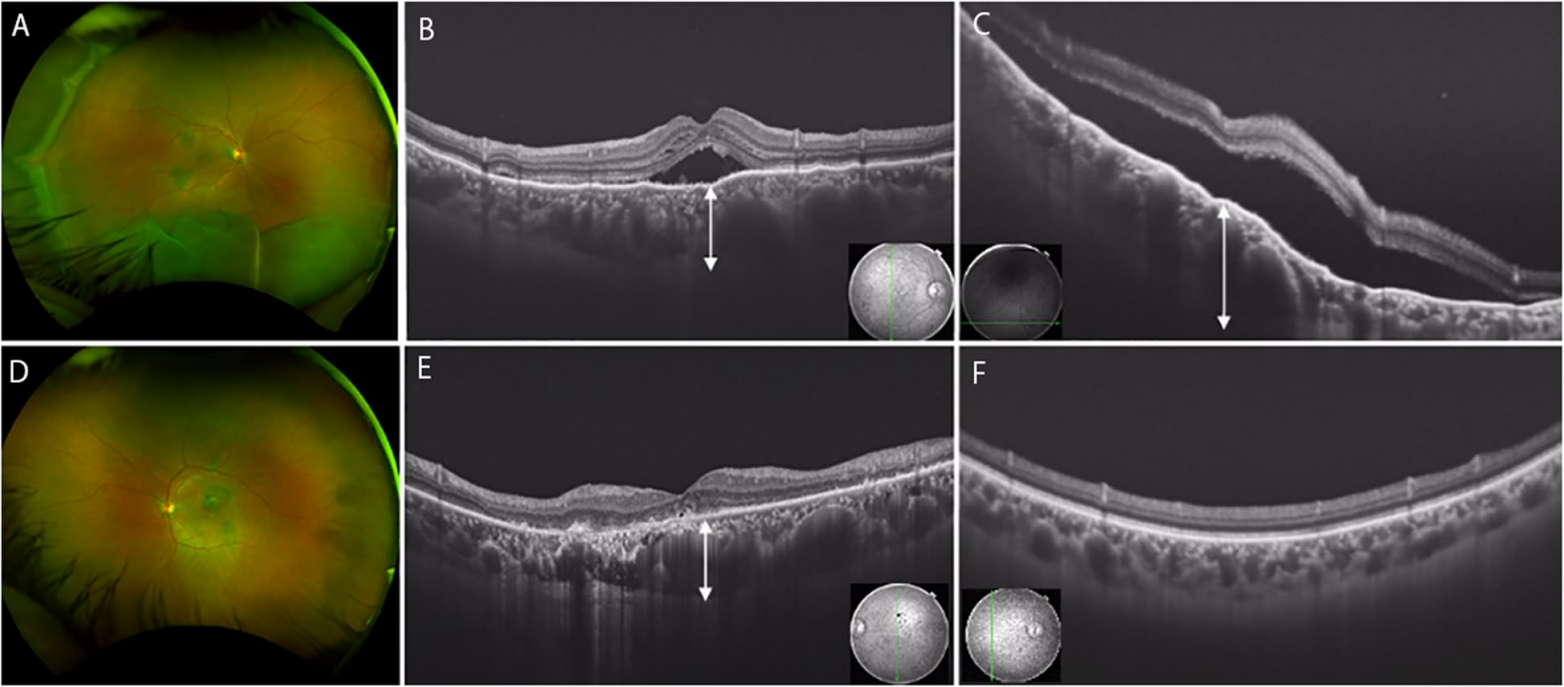
Fundus and OCT findings of the right eye (**a**-**c**) and left eye (**d**-**f**) at the initial examination. **a** The right eye shows non-rhegmatogenous retinal detachment and choroidal detachment. **b** Macular OCT shows submacular fluid and pachychoroid, with choroidal thickness of 565 μm (up-down arrow). **c** On OCT of the periphery, the choroidal thickness is 820 μm in the inferior peripheral fundus (up-down arrow). **d** Descending atrophic tract was seen in the left eye as a finding of chronic CSC. **e** Macular OCT shows pachychoroid, with choroidal thickness of 545 μm (up-down arrow). **f** OCT of the periphery shows normal choroid. OCT: optical coherence tomography, CSC: central serous chorioretinopathy

Fundus photograph of the right eye showed inferior non-rhegmatogenous retinal detachment and peripheral choroidal detachment (Fig. 1a). Descending atrophic tract was seen in the left eye as a finding of chronic CSC (Fig. 1d). In the right eye, OCT at the macula showed submacular fluid and pachychoroid, with choroidal thickness of 565 μm (Fig. 1b). In the left eye, macular OCT showed outer retinal degeneration and pachychoroid, with choroidal thickness of 545 μm (Fig. 1e). In the right eye, OCT of the inferior periphery showed retinal detachment and pachychoroid, with choroidal thickness of 820 μm (Fig. 1c). In the left eye, OCT of the peripheral fundus showed no pachychoroid (Fig. 1f).

**Fig. 2:**
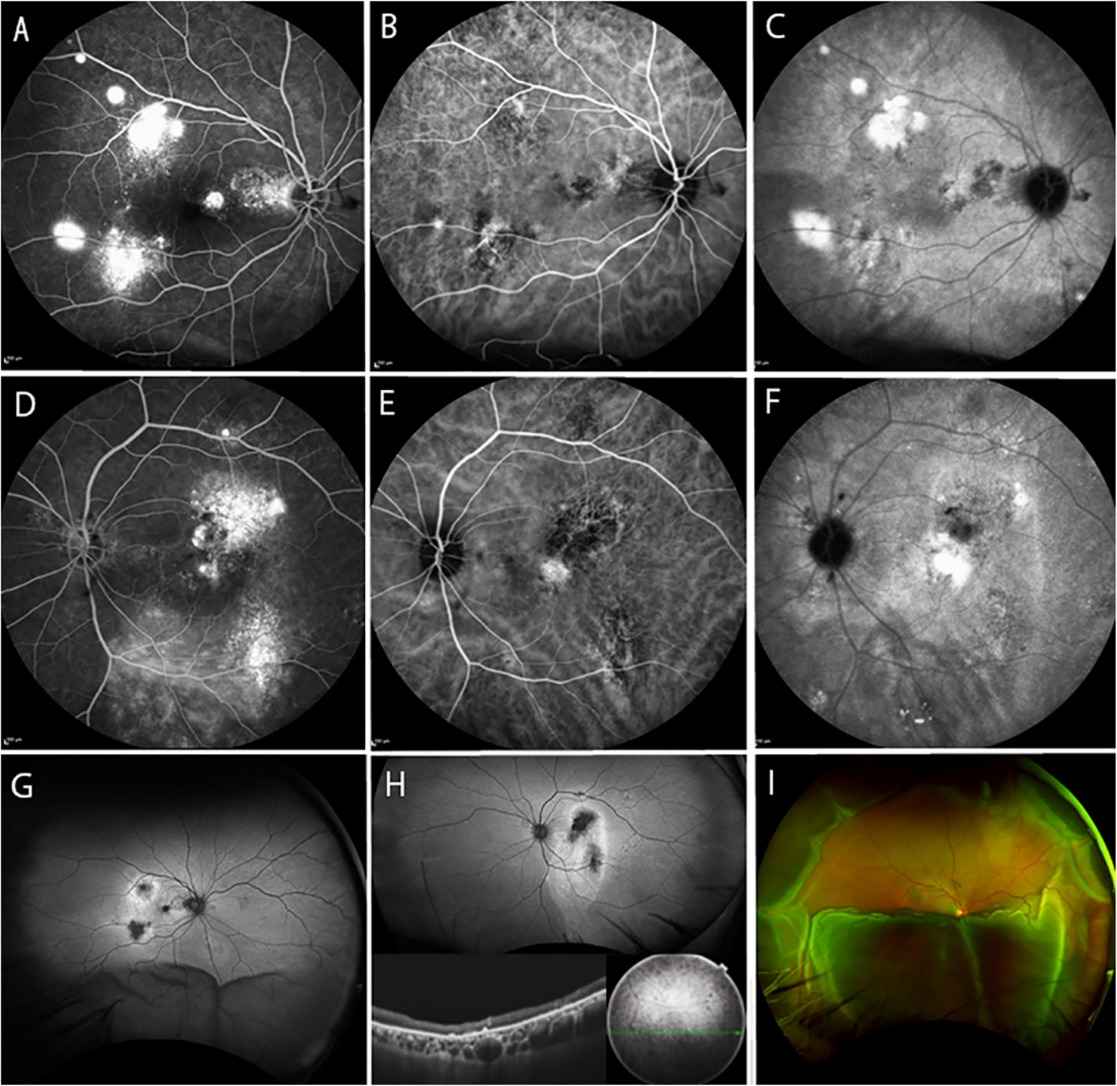
Angiographic and FAF findings at the initial examination (**a-h**) and before surgery (**i**): right eye (**a-c, g, i**) and left eye (**d-f, h**). **a, d** In the posterior pole of both eyes, late-phase FA shows window defects and mild fluorescence leakage. **b, e** In both eyes, early-phase ICGA images depict dilated choroidal vessels. **c, f** Late-phase ICGA images of both eyes depict mild choroidal hyperpermeability and mild dye leakage. **g** FAF image shows hyperautofluorescence with central hypoautofluorescence in two locations and hyperautofluorescence consistent with retinal detachment. **h** FAF imaging depicts hyperautofluorescence consistent with the descending atrophic tract from the macula. Pachychoroid is found corresponding to the descending atrophic tract. **i** One month after surgery, retinal and choroidal detachment progressed. FA: fluorescein angiography, ICGA: indocyanine green angiography, FAF: fundus autofluorescence

In the posterior pole of both eyes, late-phase FA showed window defects and mild fluorescence leakage (Fig. 2a, d), early-phase ICGA showed dilated choroidal vessels (Fig. 2b, e), and late-phase ICGA depicted mild choroidal hyperpermeability and mild dye leakage (Fig. 2c, f). Fundus autofluorescence (FAF) image of the right eye showed hyperautofluorescence with central hypoautofluorescence in two locations and hypoautofluorescence consistent with retinal detachment (Fig. 2g). FAF imaging of the left eye showed hyperautofluorescence consistent with the descending atrophic tract from the macula and two central areas of hypoautofluorescence. Pachychoroid was found corresponding to the descending atrophic tract (Fig. 2h). The left eye was diagnosed with chronic CSC. Based on the presence of submacular fluid and pachychoroid, the right eye was initially suspected of having severe CSC and treated with photodynamic therapy for the two fluorescent leaks. However, fluorescence leakage and pachychoroid did not improve, and retinal detachment and choroidal detachment spread further. Since peripheral pachychoroid was found only in the right eye, the right eye was finally diagnosed as UES rather than chronic CSC. The patient was followed conservatively because of good visual acuity. Two months later, retinal and choroidal detachment progressed in the right eye (Fig. 2i), and visual acuity decreased to 12/200.

**Fig. 3:**
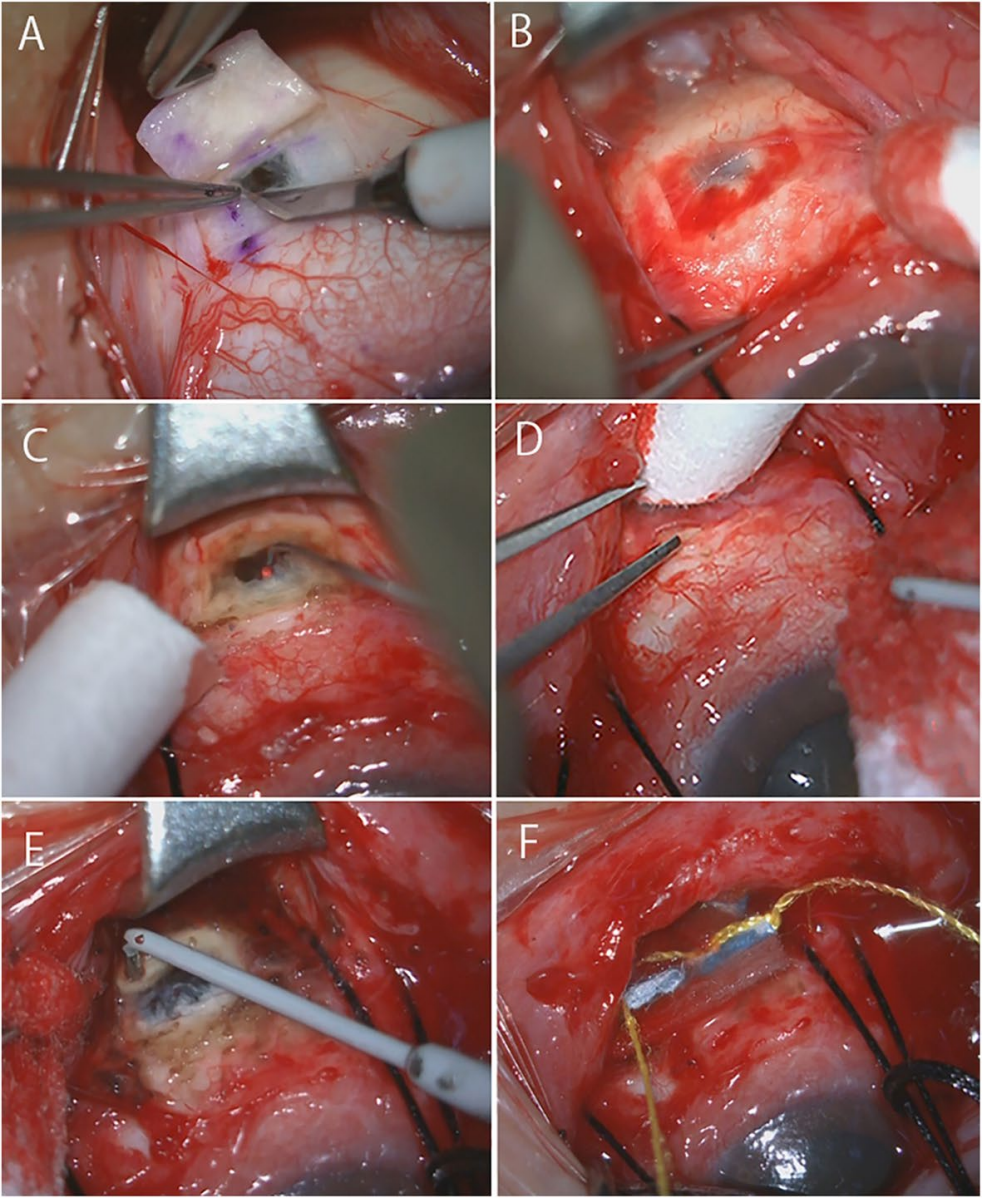
Intraoperative photographs at the initial surgery (**a**), second surgery (**b, c**), and third surgery (**d-f**). **a** Full-thickness sclerectomies were performed. At the end of surgery, the scleral flap is resected. **b** At the second surgery, the scleral incision is adhered to tissue of the Tenon’s capsule tissue and connective tissue regeneration is observed. **c** A full-thickness sclerotomy is made and subretinal fluid is drained using 532 nm intraocular laser probe. **d** At the third surgery after the second recurrence, complete connective tissue regeneration at the surgical site is observed. **e** Tissue of the Tenon’s capsule is removed, a full thickness sclerectomy is performed, and the surrounding sclera is coagulated with diathermy. **f** Finally, mitomycin-C is applied for 3 mins. UES: uveal effusion syndrome

Full-thickness sclerectomies were performed in the right eye. In each quadrant, a 2 × 2 mm full-thickness sclerectomy with a 4 × 4 mm partial scleral flap was made at 8 mm from the limbus. At the end of the surgery, the four scleral flaps were resected (Fig. 3a). Since there was no scleral thickening and no glycosaminoglycan deposits on histopathological examination, the diagnosis was confirmed to be type 3 UES. Postoperatively, choroidal detachment was resolved gradually, but the bullous retinal detachment did not improve even after 1 month. Therefore, reoperation was performed.

In the second surgery, the sclerectomy sites were found to be adhered to the tissue of Tenon’s capsule, and connective tissue regeneration was observed (Fig. 3b). Therefore, full-thickness sclerectomies were performed in the two inferior quadrants. Then, subretinal and choroidal fluid was drained by irradiating the full-thickness sclerectomy site with an intraocular laser probe (532 nm laser) (Fig. 3c). When the sclera was compressed, a large amount of yellow, highly viscous retinochoroidal fluid was drained. Because of the low IOP, saline was injected into the vitreous to correct the IOP. The retinal and choroidal detachment disappeared when the fundus was observed immediately afterwards. After the second surgery, the retinal detachment and choroidal detachment were almost resolved, and visual acuity improved to 12/20. However, 1 month after surgery, retinal detachment exacerbated and generalized choroidal detachment also appeared, and visual acuity decreased to 8/20.

In the third surgery, the scleral site had adhered to the Tenon’s capsule with complete connective tissue regeneration (Fig. 3d). For this reason, the Tenon’s capsule tissue was removed, full-thickness sclerectomies in the two inferior quadrants were performed, the surrounding sclera was coagulated with diathermy (Fig. 3e), and 0.04% mitomycin-C was applied for 3 min (Fig. 3f).

**Fig. 4:**
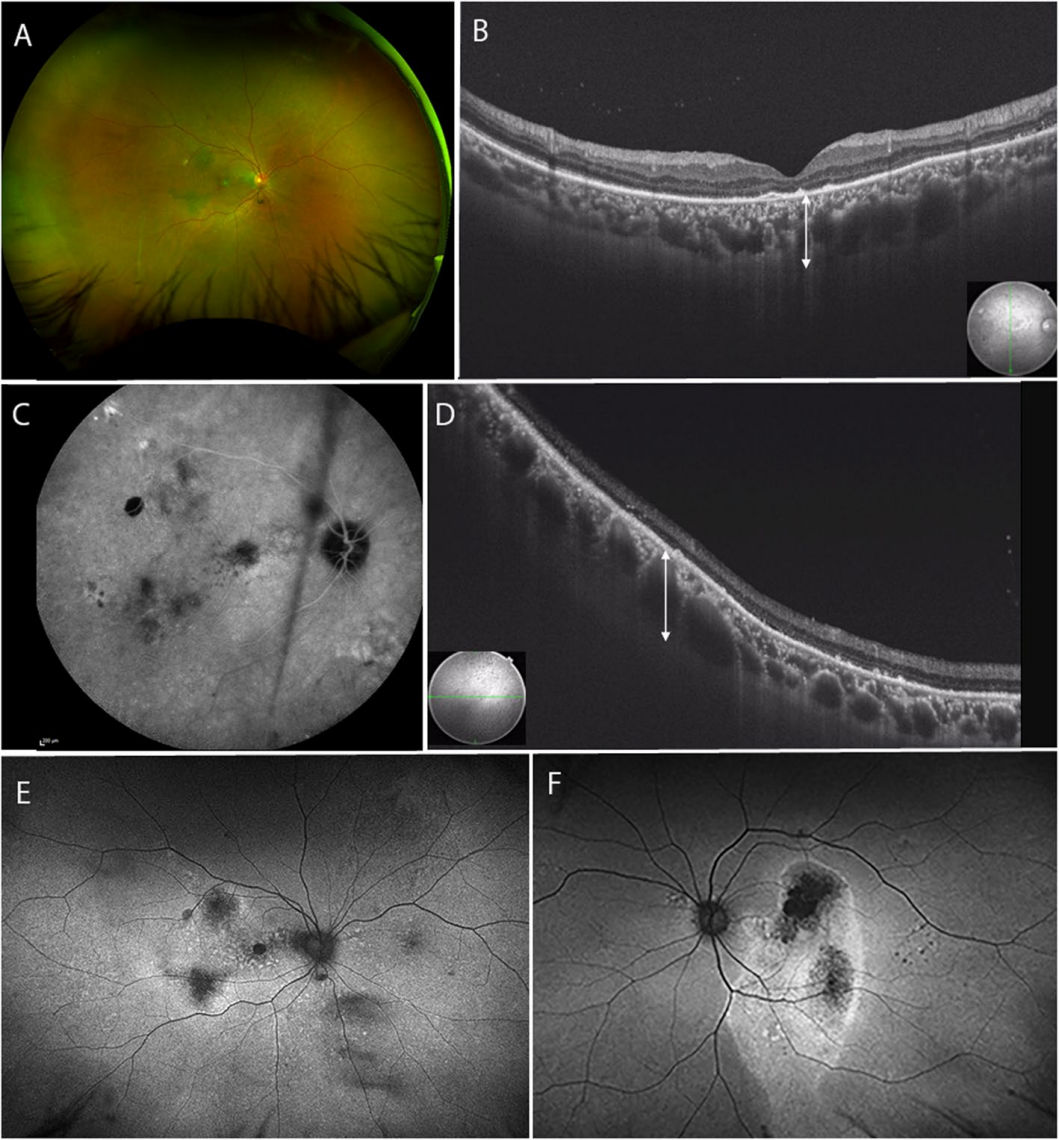
Fundus findings after the third surgery. **a** One month after surgery, retinal and choroidal detachment have disappeared. **b** On OCT, macular choroidal thickness has decreased to 352 μm (up-down arrow). **c** In late-phase ICGA, choroidal hyperpermeability and dye leakage have improved. **d** On OCT, the choroidal thickness in the inferior peripheral fundus has decreased to 554 μm (up-down arrow). **e** FAF image shows hyperautofluorescent spots over a large area of the fundus. **f** FAF image depicts hyperautofluorescence consistent with the descending atrophic tract from the macula. ICGA: indocyanine green angiography, OCT: optical coherence tomography, FAF: fundus autofluorescence

One month after surgery, retinal and choroidal detachment disappeared (Fig. 4a), and visual acuity improved to 8/20. In late-phase ICGA, choroidal hyperpermeability and dye leakage improved (Fig. 4c). The pachychoroid in the macula and peripheral fundus decreased, and choroidal thickness decreased to 352 μm in the macula (Fig. 4b) and 554 μm in the inferior periphery of the fundus (Fig. 4d). FAF imaging of the right eye showed hyperautofluorescent spots over a large area of the fundus (Fig. 4e). FAF imaging of the left eye showed hyperautofluorescence corresponding to the descending atrophic tract from the macula, as in the initial examination (Fig. 4f).

## Discussion and conclusions

Uyama et al. [4] classified two eyes that did not show scleral thickening or glycosaminoglycan deposition as type 3 UES, and subscleral sclerectomy was not effective for these eyes. However, reoperation was not performed, and the cause of the ineffective subscleral sclerectomies was unknown. In the present case, the left eye had undergone conventional focal photocoagulation twice in the past and fundus examination depicted a descending atrophic tract, which is known to be a characteristic finding of chronic CSC [6]. Thus, the left eye was diagnosed with chronic CSC.

The right eye with submacular fluid and choroid was initially suspected of having severe CSC. Photodynamic therapy has been reported to be effective not only for chronic CSC [7] but also for CSC with choroidal detachment [8]. We therefore performed photodynamic therapy for the fluorescent leakage in the right eye. However, fluorescence leakage, pachychoroid and retinal detachment did not improve, while retinal and choroidal detachment spread further. Furthermore, pachychoroid in peripheral fundus was observed only in the right eye, and it has been reported that peripheral pachychoroid is not seen in CSC [9]. Based on the above findings, UES was diagnosed in the right eye. In addition, full-thickness sclerectomies combined with mitomycin-C improved the retinal and choroidal detachment in the right eye. Therefore, we believe that the clinical findings in the right eye are in favor of UES rather than CSC. However, overlap of clinical findings between UES and variant CSC has been reported [10, 11]. The differentiation between UES and variant CSC remains unclear. It is necessary to study a large number of UES cases to further investigate the relationship of UES with CSC.

Drainage of subretinal and choroidal fluid, which has been reported to be effective for UES [12], was performed simultaneously with full-thickness sclerectomy, but only transient effect was obtained. Connective tissue regeneration at the sclerectomy sites was speculated to be the reason for the ineffectiveness of subretinal and choroidal fluid drainage and full-thickness sclerectomies. Based on this hypothesis, revision surgery was conducted using full-thickness sclerectomies with mitomycin-C to inhibit connective tissue regeneration, and favorable outcomes were obtained. This clearly indicates that type 3 UES has scleral abnormalities. Since type 3 UES has mildly thickened or normal sclera, we believe that mitomycin-C combined with sclerectomies [13] or ultrasound-guided sclerectomies [14] would be more effective than subscleral sclerotomies or simple sclerectomies.

The eye with type 3 UES and the other with CSC had the following common manifestations in the posterior pole: window defects and mild fluorescence leakage in FA, dilated choroidal vessels, mild choroidal hyperpermeability, and dye leakage in ICGA. The common denominator of both type 3 UES and CSC is the presence of dilated choroidal vessels and choroidal thickening on OCT at the posterior pole, suggesting that both are pachychoroid spectrum diseases [5, 9, 15]. Both diseases are also similar in having no glycosaminoglycan deposition in the scleral tissue.

However, there are some differences between type 3 UES and CSC. In FAF imaging, focal thickening of the retinal pigment epithelium after absorption of retinal detachment can be seen as hyperautofluorescent spots [16], which facilitates evaluation of lesion size. Type 3 UES and CSC differ in lesion size. Pachychoroid, which causes retinal detachment, is present only in the macula in CSC [9], but is present from the macula to the peripheral fundus in type 3 UES. Consequently, UES lesion occupies a large area of the fundus, while CSC lesion is confined to an area centered on the macula. In type 3 UES, the preoperative choroidal thickness was 565 μm in the macula and 820 μm in the peripheral fundus, suggesting that the peripheral pachychoroid is the main cause of retinal and choroidal detachment in type 3 UES.

Type 3 UES is considered to have no scleral thickening. Histological measurements of scleral thickness at the posterior pole averaged 0.95 ± 0.18 mm in healthy subjects (mean age 62 years, axial length ≤ 26 mm) [17]. Measurements of unfixed resected sclera in patients with uveal effusion syndrome (mean age 64 years) averaged 1.3 mm (1.3–1.4 mm) in patients without intraoperative scleral thickening and 2.3 (1.5–2.9 mm) in those with intraoperative scleral thickening [18]. Therefore, although scleral thickness observed during surgery may appear normal, the sclera may be thicker than that in healthy individuals.

Imanaga et al. [19] measured scleral thickness by anterior segment OCT at 6 mm posterior to the scleral spur in 4 directions, and reported that scleral thickness in eyes with CSC was significantly thicker than that in healthy subjects by 20–50 μm at each site. Based on these results, they proposed that thick sclera may be the etiology of CSC.

In conclusion, this case demonstrated pachychoroid in the posterior pole as the common clinical manifestation of type 3 UES and CSC, although the extensive pachychoroid in the peripheral fundus in type 3 UES may have caused retinal and choroidal detachment. Full-thickness sclerectomies with mitomycin-C improved pachychoroid in the peripheral fundus with resolution of retinal and choroidal detachment, clearly indicating that the sclera was the main cause of type 3 UES.

## Data Availability

Not applicable.

## Abbreviations

CSC: central serous chorioretinopathy
FA: fluorescein angiography
FAF: fundus autofluorescence
ICGA: indocyanine green angiography
IOP: intraocular pressure
OCT: optical coherence tomography
UES: uveal effusion syndrome
